# Exposome Versus Genome in HS: How Do We Currently Explain Where Disease Arises from?

**DOI:** 10.3390/jcm15145332

**Published:** 2026-07-08

**Authors:** Emily G. Summers, Olivia D. Perez, Christopher J. Sayed, Lynn Petukhova

**Affiliations:** 1 Department of Dermatology, University of North Carolina at Chapel Hill School of Medicine, Chapel Hill, NC 27516, USA; emily_summers@med.unc.edu; 2 The Ronald O. Perelman Department of Dermatology, New York University Langone Health, New York, NY 10016, USA; 3 Department of Population Health, New York University Langone Health, New York, NY 10016, USA

**Keywords:** hidradenitis suppurativa, genetics, genome-wide association study, polygenic risk score, environmental factors, obesity, smoking, microbiome, diet, plastic consumption

## Abstract

Hidradenitis suppurativa (HS) is a chronic inflammatory skin disease with marked clinical heterogeneity and a multifactorial pathogenesis. Environmental and lifestyle factors, including obesity, tobacco exposure, microbiome dysbiosis, dietary patterns, and plastic-associated endocrine disruptors, have all been linked to HS risk or disease severity. However, these exposures alone do not fully explain why only some individuals develop HS, why age at onset and severity vary substantially, or why disease occurs in patients without major identifiable environmental burden. In parallel, genetic studies have demonstrated that inherited susceptibility is a central component of HS pathogenesis. Rare loss-of-function variants in γ-secretase complex genes cause a small subset of familial, autosomal dominant HS, while genome-wide association studies have shown that population-level risk implicates pathways involved in epithelial differentiation, follicular biology, immune signaling, and cutaneous inflammation. Recent work further suggests that common and rare genetic risk may converge on shared biological mechanisms, including γ-secretase-related signaling. While nature vs. nurture arguments dichotomize genetic constitution and environmental exposures, current evidence supports a model in which genetic susceptibility interacts with environmental exposures to shape HS risk, clinical expression, and disease progression. In this review, we examine the evidence supporting both exposomic and genomic contributions to HS and argue that the disease is best understood as arising from their intersection rather than from either domain alone.

## 1. Introduction

Hidradenitis suppurativa (HS) is a chronic inflammatory skin disease characterized by recurrent nodules, abscesses, tunnels, and scarring in intertriginous areas [1]. Despite growing recognition of its clinical burden, the fundamental question of why HS develops remains unsettled. Is HS primarily a disease of environmental exposure, driven by factors such as obesity, smoking, diet, microbiome disruption, and plastic-associated endocrine disruptors? Or is it fundamentally a genetic disease, in which inherited susceptibility determines who develops the disease, when it begins, and how severe it becomes? Current evidence can be marshaled in support of both views, making HS an especially compelling model for examining the relative contributions of exposome and genome.

The argument for the exposome is intuitive and clinically persuasive. HS is strongly associated with obesity and tobacco use, and both are linked to greater disease severity in multiple cohorts [2]. Additional environmental factors—including microbiome dysbiosis, dietary patterns, and emerging evidence related to plastic-associated endocrine-disrupting chemicals—have also been implicated in disease pathogenesis [3,4,5,6]. These observations align with the perception that HS prevalence may be rising, particularly in Western populations, raising the possibility that changing environmental exposures are contributing to disease expression in ways that genetic variation alone cannot explain [7]. From this perspective, HS may be viewed as a disease that emerges when inflammatory, metabolic, microbial, and mechanical insults converge on susceptible skin.

Yet the counterargument is equally strong: environmental associations do not explain why many heavily exposed individuals never develop HS, while others develop severe disease in the absence of obvious environmental burden. Familial clustering is common, with a positive family history reported in approximately 30–60% of patients, and twin studies estimate heritability as high as 74–80%, strongly supporting an inherited component to disease susceptibility [8,9,10,11]. Rare loss-of-function variants in γ-secretase complex genes such as *NCSTN*, *PSENEN*, and *PSEN1* establish a Mendelian form of HS in a subset of families, while more recent genome-wide association studies (GWASs) demonstrate that polygenic risk better aligns with the population burden [12,13,14,15]. These common-variant studies increasingly implicate pathways involved in epithelial differentiation, follicular biology, immune signaling, and cell-type-specific regulatory programs, providing mechanistic insight that environmental association studies alone often cannot [16,17,18,19].

This creates the central tension in HS pathogenesis: the exposome offers plausible and clinically observable triggers, whereas the genome offers a biologically grounded explanation for differential susceptibility. Framed this way, the debate is not simply whether HS is environmental or genetic, but which domain provides the more fundamental explanation for where disease arises from. Environmental models help explain disease modulation and temporal trends, whereas genetic models help explain familial aggregation, early-onset disease, and interindividual heterogeneity in risk and phenotype.

At the same time, treating these explanations as mutually exclusive is likely misleading. Environmental exposures act through biologic pathways that are genetically encoded, and genetic susceptibility may shape how individuals respond to inflammatory, microbial, hormonal, or xenobiotic stressors. Emerging data suggest that rare and common HS risk variants converge on pathways that may also be environmentally responsive, including γ-secretase signaling, microbial response pathways, and xenobiotic metabolism [10,20,21]. These observations support a more integrated model in which inherited susceptibility establishes the threshold for disease, while environmental exposures influence whether, when, and how that threshold is crossed.

In this review, we adopt a point–counterpoint framework to examine the evidence on both sides of this debate. We first consider the case that environmental factors are foundational in HS development and severity, then review the evidence that inherited genetic variation forms a cornerstone of HS pathogenesis. We conclude by arguing that HS is best understood not as a purely environmental or purely genetic disease, but as one that arises from the interaction between the two.

## 2. Point: Environmental Factors Are Foundational in HS Development and Disease Severity

Although family history is strongly associated with HS, some patients develop this disease without a family history. Environmental factors have been shown to be associated with HS. Exposures such as smoking and obesity increase overall inflammation in the body and are the environmental factors most strongly associated with this disease [2]. Other environmental factors linked to HS are microbiome dysregulation, dietary factors, and plastic consumption, as seen in Table 1.

### 2.1. Obesity

Increased body mass index (BMI) is associated with increased HS disease severity [22]. Not all patients with HS have an increased BMI; however, the current literature reveals a strong association. Possible causes for this association are multi-faceted, as increased BMI is associated with increased adiposity, hormonal imbalances, inflammatory markers, mechanical stress, and metabolic abnormalities, all of which are associated with HS.

For most patients, increased BMI results in increased adipose tissue. Adipose tissue has been shown to produce active estrogens in premenopausal women and store these hormones within this tissue [23]. In addition, cutaneous conversion of testosterone to estrogen occurs in subcutaneous fat [26]. Increased estrogen and other sex hormones may influence the homeostasis of the skin and contribute to infundibular hyperkeratinization of the hair follicle [28]. HS is thought to partially be a result of dysregulated follicular stem cells, leading to epidermal hyperkeratosis in the hair follicles and resulting in dermal epithelial wall rupture with subsequent inflammatory response to follicular contents [28]. Obesity and metabolic abnormalities increase pro-inflammatory adipokines in this subcutaneous adipose tissue, possibly contributing to the inflammatory etiology of HS [24].

Lastly, friction or mechanical stress resulting in HS disease flares has been frequently reported by patients as a trigger [29]. The newer literature suggests that mechanical stress in the setting of inflammation may contribute to the development of lesions [25]. Taken together, obesity can lead to mechanical stress and multiple inflammatory pathways that may contribute to the pathogenesis of HS lesions, especially in intertriginous areas.

### 2.2. Smoking

Similar to obesity, increased smoking pack-years is also linked to greater HS disease severity [22]. Studies show that nicotine, along with other chemicals in tobacco products, promotes pro-inflammatory cytokines that can be found in HS lesions [27]. In addition, nicotine has been found to promote colonization of Staphylococcus aureus in intertriginous areas, which may result in dysbiosis that influences disease activity [30]. Increasing Staphylococcus aureus has been found to be significantly more frequent in patients with HS Hurley stage III compared to patients with stages I and II [31]. Though data quality is limited, there is suggestive evidence that smoking quantity and duration may be linked to worse disease activity and lower response to treatment, though the impact of smoking cessation on short-term disease activity is unclear. There is also evidence that specific disease phenotypes, in particular the scarring folliculitis phenotype, may be more highly linked to increased rates of tobacco use than the more common “regular” phenotype [32,33,34].

### 2.3. Microbiome

A patient’s microbiome reflects both the intestines or gut and the skin. The impact of microbiome dysregulation or altered host–microbiome interactions in HS pathogenesis is poorly understood; however, there is evidence of dysbiosis between HS and non-HS patients in HS skinfolds. Patients with HS were found to have an increased abundance of anaerobes, predominantly Prevotella, in addition to Actinomyces, Campylobacter ureolyticus, and Mobiluncus, with a lower concentration of the skin’s major microbiome bacterium, Staphylococcus epidermidis [3]. This dysbiosis alters the skin barrier and may promote the formation of HS lesions. In addition, gut microbiota alterations have been shown in HS patients, with a higher abundance of Rominococcus gnavus in the fecal microbiome [35]. This finding is also reported in patients with Crohn’s disease, possibly explaining the strong association of irritable bowel disease with HS [35].

The role of the microbiome is further supported by the positive response to antibiotic treatment. Many antibiotics seem to impact disease activity in HS, though results are inconsistent, and regimens with highly variable antimicrobial spectra have been reported [36,37]. The results likely reflect impact on the microbiome and subsequent host response rather than an anti-inflammatory effect, given that very broad-spectrum antibiotics like ertapenem may have the most dramatic treatment effect [38]. Loss of response after stopping treatment is typical since the microbiome shifts closer to baseline once treatment is stopped.

### 2.4. Dietary Factors

Dietary factors leading to HS development are a topic of debate. Nutritional research is challenging to perform; however, the current literature links specific foods with HS disease severity. Below, we explore common foods that have been associated with HS development, including ultra-processed foods, simple carbohydrates, and dairy.

#### 2.4.1. Ultra-Processed Foods

HS prevalence and ultra-processed food consumption rates are highest among Non-Hispanic Black people, whereas those of Hispanic ethnicity have the lowest prevalence of HS and lowest consumption of ultra-processed foods [4,39,40]. Hispanic populations also have the highest rates of minimally processed food consumption in the US [39]. With these findings and the fact that both groups have higher relative obesity rates compared to white people, this poses the question of whether ultra-processed foods are associated with HS pathogenesis [4]. Researchers found ultra-processed food consumption was strongly correlated with HS prevalence among Non-Hispanic Black individuals and all races in patients aged 20–39 years old [4]. They also found a significant increase in Google searches for HS that strongly correlated with an increase in ultra-processed food consumption [4].

#### 2.4.2. Simple Carbohydrates and Dairy

Simple carbohydrates and dairy have been associated with the initial stages of HS pathogenesis, where the hair follicle occludes and ruptures [5]. This is thought to occur due to the breakdown products of simple carbohydrates, which increase insulin and insulin-like growth factor, resulting in the activation of the mechanistic target of rapamycin (mTOR) pathway and androgen receptors, leading to hyperkeratinization and follicular occlusion [5,41]. Additionally, mTOR is involved in the regulation of T helper cells that are associated with HS progression [5,42].

### 2.5. Plastic Consumption Associated with Endocrine Disruptors

Plastic consumption has continued to rise since the early 2000s [43]. Health outcomes related to the increase in microplastic exposure are an area of high interest and rapidly evolving understanding. Preliminary studies reveal potential linkages between plastic consumption and HS pathogenesis. Williams et al. reported that HS patients have elevated levels of plastic-associated endocrine-disrupting chemicals (p-EDs) in their skin [6]. They found these p-EDs inhibit Nicastrin (NCSTN) protein expression, promoting inflammation. NCSTN regulates immune signaling in fibroblasts and is a key component of the gamma-secretase complex, which is implicated in a small proportion of familial HS cases. This decrease in Nicastrin function may partially explain the rise in HS cases, as ultra-processed foods and microplastic exposure have increased in the last few decades. It may also explain the mechanistic link between familial cases and sporadic cases through impaired γ-secretase activity [6]. Future studies are needed to validate these newer associations.

### 2.6. Summary of Environmental Findings and Future Directions

Taken together, multiple environmental factors are associated with HS severity and disease development. It is challenging to determine the complete pathogenesis of this chronic disease. We outline several factors that are prevalent in the literature, including obesity, smoking, microbiome, dietary factors, and plastic consumption. Limitations include the lack of research studies establishing causal relationships between these environmental factors and HS. Additionally, the strength of evidence supporting these factors varies, as the associations between HS and obesity and smoking are well established, whereas the associations with microbiome, dietary factors, and plastic consumption are more preliminary.

Despite there being higher rates of HS, family history is still very common and reported in up to 50% of patients in some cohorts [9]. This suggests there was likely historical under-reporting related to missed and delayed diagnoses. In addition, there is a strong association between HS and smoking. Rates of HS have continued to rise even though rates of smoking are on the decline in the US [44]. Ultimately, genetic factors may lower the threshold for developing the disease in the face of environmental contributions. Taken together, both genetic and environmental factors probably influence disease development.

## 3. Counterpoint: Inherited Genetic Variants Form a Cornerstone of HS Pathogenesis

Although environmental and lifestyle factors such as elevated BMI and tobacco use are strongly associated with HS, these exposures alone are insufficient to explain disease occurrence. Specifically, a recent meta-analysis of 25 studies found that smoking increases the odds of HS by fourfold [27], whereas polygenic risk increases HS risk eightfold [19]. There is a correlation between early-onset HS and stronger genetic susceptibility and more widespread involvement [45], highlighting a need to elucidate genetic pathways. Importantly, environmental risk factors provide limited mechanistic insight, whereas genetic risk variants lead directly to knowledge about genes, pathways, and cell types and also facilitate the identification of drug targets.

### 3.1. A Primer on Genetic Methodologies

HS has a substantial genetic component [46,47], as demonstrated by family-based linkage studies, candidate gene analyses, sequencing-based approaches, and genome-wide association studies (GWASs). Here, we review genetic methodologies for the practicing clinician to provide essential context for interpreting the genetic evidence reviewed below. These methodologies are summarized in Table 2.

Common diseases such as cardiovascular disease, obesity, and diabetes arise from the combined effects of many genetic variants that are common in the population, whereas monogenic disorders (e.g., sickle cell anemia) result from pathogenic variants in a single gene that are sufficient to cause disease [48]. HS spans both paradigms. Rare monogenic forms have been described in select families [12], but most disease risk in the population is explained by polygenic inheritance [15].

Linkage analysis is a family-based approach in which genomes from affected relatives are scanned for chromosomal regions that co-segregate with disease across generations [49]. This method is most powerful in large pedigrees with clear inheritance patterns and was instrumental in establishing autosomal dominant transmission of rare loss-of-function γ-secretase variants in a subset of familial HS cases [12].

Candidate gene studies test predefined genes selected based on biological plausibility or syndromic co-presentation [50]. While efficient and hypothesis-driven, these studies are inherently limited by prior knowledge and do not provide genome-wide statistical evidence [51]. As a result, candidate gene findings often rely on case reports or small series and require independent replication and functional validation [52]. This approach has been instrumental in characterizing HS as an auto-inflammatory disease and laying the foundation to investigate inborn errors of immunity (IEI) in HS [53,54,55].

Whole-exome sequencing (WES) and whole-genome sequencing (WGS) enable unbiased interrogation of genetic variation across all protein-coding regions or the entire genome, respectively [56]. These approaches are particularly useful for identifying rare variants in familial or early-onset disease but require large sample sizes and appropriate statistical frameworks to implicate genes in cohorts of unrelated individuals [57].

In contrast, GWASs examine common genetic variants across the genome in large case–control cohorts to identify loci associated with disease susceptibility [58]. GWASs are designed to detect variants with modest individual effects that collectively contribute to disease risk and facilitate the development of polygenic risk scores (PRSs), instruments that can be used for risk stratification [59]. As cohort sizes increase, HS GWASs provide increasing power to identify novel risk loci and genetic architecture that is shared with comorbid conditions [18,19,60,61]. The development of an HS PRS also holds promise for reducing HS diagnostic delays [19]. However, this area remains at an early stage, and the clinical utility of an HS PRS is currently uncertain.

Together, these complementary methodologies define a spectrum of disease alleles ranging from rare, high-impact variants to common, low-effect alleles. Statistical evidence generated by any of these methods provides a starting point for assessing causality; however, additional criteria must be met to establish confidence that implicated genes and variants exert meaningful biological and clinical effects [57]. Statistical association determines whether genetic variation is associated with disease beyond chance within a given study design. Replication across independent cohorts or populations strengthens confidence in the robustness and generalizability of these findings. Functional validation further evaluates whether implicated variants plausibly alter biological pathways relevant to disease pathogenesis. The sections below review how each methodological approach has contributed to the current understanding of HS genetics, highlighting these levels of evidence to contextualize the strength and limitations of reported genetic associations, as summarized in Table 3.

### 3.2. The Genetic Basis of Hidradenitis Suppurativa

Familial aggregation provides an indication that inherited genetic factors contribute to disease susceptibility, even when environmental and lifestyle exposures are shared. HS has long been recognized as a condition with familial clustering, supporting a substantial genetic contribution to disease risk. Surveys across multiple HS cohorts report that approximately 30–60% of HS patients have a positive family history. In a case series of 618 French HS patients, 35% of participants reported at least one affected relative [8]. Similarly, in a cohort of 676 HS patients at the University of North Carolina, 57.5% reported HS in a first- or second-degree relative [9]. Twin studies further support a strong inherited component, with estimated heritability of 80% in a Danish cohort [10] and 74% in a Dutch cohort [11].

### 3.3. Rare Variant Contributions to HS

Rare variant studies rely primarily on statistical enrichment within families, candidate gene sequencing in participants with extreme phenotypes, or population-level testing for genes that have an excess burden of rare damaging variants. The strength of evidence comes from segregation in families, replication across unrelated pedigrees, constraint in reference populations (purifying selection) and functional consequence (Table 3).

#### 3.3.1. γ-Secretase Complex Genes: The Strongest Rare-Variant Signal in HS

Landmark family-based linkage and sequencing studies identified statistically significant co-segregation of rare pathogenic variants in *NCSTN*, *PSENEN*, and *PSEN1*, establishing γ-secretase loss-of-function as causal in autosomal dominant, Mendelian forms of HS (OMIM 142690) [12,13,14].

A second line of evidence comes from replication. Pathogenic variants in γ-secretase genes have been independently reported across multiple pedigrees and populations, consistently demonstrating autosomal dominant inheritance [62,63]. Collectively, these studies establish γ-secretase genes as validated HS risk genes, although they account for only a small fraction of total HS cases, affecting 2–3% of HS participants [64,65].

A third line of evidence is provided by population genetics. In large reference datasets, γ-secretase genes show strong signatures of purifying selection, with loss-of-function variants being exceedingly rare, supporting the pathogenicity of such variants when observed in HS patients [66,67].

In contrast, functional genomic evidence remains limited. In vitro studies suggest that *NCSTN* loss of function alters keratinocyte proliferation and differentiation through dysregulated Notch signaling and secondary effects on PI3K/AKT signaling [68]. However, these functional findings have not been widely replicated and remain incompletely integrated with human genetic data, representing an important area for future investigation.

#### 3.3.2. Candidate Gene Studies of Rare Variants

Candidate gene studies test a priori hypotheses and evaluate predefined genes selected on the basis of syndromic co-presentation and shared disease pathways. In contrast to γ-secretase genes, most candidate gene associations in HS are supported by limited statistical evidence and rely largely on case reports or small case series (Table 3).

Genes that cause ectodermal dysplasias (EDs) have been implicated by rare variants in sequencing studies of rare ED syndromes that co-present with HS. For example, a pathogenic variant in *GJB2*, encoding a gap junction protein, has been described in patients with keratitis–ichthyosis–deafness (KID) syndrome (OMIM #148210). These patients also exhibited the follicular occlusion triad (HS, dissecting cellulitis, acne conglobata) [69,70,71,72,73,74]. Two cases with rare variants in *IRF6* resulting in ED co-presenting with recurrent abscesses in the axilla, groin, and gluteal areas were reported [75]. IRF6 is broadly expressed in embryonic and adult tissues, and pathogenic variants in *IRF6* result in Van der Woude syndrome 1 and popliteal pterygium syndrome (OMIM: 119300) [75,76]. In addition, rare variants of KDF1 have been associated with an autosomal dominant form of ED and recurrent skin abscesses [77,78].

Syndromic co-presentation also provided a rationale for candidate gene studies that implicated *PSTPIP1* in HS [79]. PAPA syndrome (OMIM 604416) is a monogenic auto-inflammatory disorder that is caused by rare pathogenic variants in *PSTPIP1* and that sometimes co-presents with HS [80,81]. Candidate gene sequencing in a cohort of participants with HS and PAPA syndrome discovered promoter repeat variants [53]. However, among all published cases, only one reported variant meets pathogenicity criteria [81]. More recently, *PSTPIP1* was shown to have a significantly increased number of rare missense variants in a cohort of 117 individuals with HS compared to a healthy control population [82].

The implication of *PSTPIP1* in HS led to the proposition that HS is an auto-inflammatory keratinization disease [83] and opened the door to investigating other immune-mediated Mendelian diseases. Auto-inflammatory diseases represent only one subclass of inborn errors of immunity (IEI). HS co-presentation has also been reported across diverse IEI categories, including defects in humoral immunity (common variable immunodeficiency and mannose-binding lectin deficiency; *IKBKB*), interferon signaling (*STAT1* gain-of-function; *STAT3* loss-of-function), ubiquitin-mediated cell death pathways (XIAP deficiency), and inflammatory metabolism (LACC1) [54,55]. These preliminary findings suggest that HS is an underrecognized component of IEI. There are important clinical implications to identifying undiagnosed IEI in HS cohorts [84]. Currently, however, evidence for these associations is derived primarily from case reports and small case series [55].

The principal limitation of candidate gene studies in HS is the absence of large-scale sequencing in unselected HS cohorts, coupled with gene-level statistical enrichment analyses. Despite the availability of well-established functional assays for many IEIs, systematic functional genomic studies in HS patient samples remain rare, limiting mechanistic interpretation of these genetic observations.

A small number of whole-exome sequencing (WES) studies have been reported [85,86,87]; however, these studies were underpowered for genetic discovery due to modest sample sizes, absence of appropriate gene-level statistical testing, and lack of independent replication.

### 3.4. Common Variant Architecture of HS

HS GWASs are rapidly expanding our knowledge of common HS risk variants. GWASs detect modest-effect alleles across the genome and gain power as cohort size increases, enabling both locus discovery and identification of shared genetic architecture [88]. To date, multiple GWASs have been conducted in HS, as summarized in Table 4.

The first HS GWAS was conducted in 720 cases from the University of North Carolina HS ProCARE cohort and meta-analyzed with data from three large biobanks (UK Biobank, FinnGen, and BioVU), yielding approximately 1640 cases in the discovery phase and 290 cases for replication [60]. This study identified two genome-wide significant loci near *SOX9* (chromosome 17) and *KLF5* (chromosome 13) [60]. Risk variants at these loci localize to keratinocyte regulatory elements, implicating transcriptional dysregulation of genes involved in epidermal differentiation, follicular biology, and cutaneous inflammation in HS pathogenesis [16,17].

Subsequent GWASs have substantially expanded the number of implicated loci. A 2025 study of 4814 HS cases identified eight independently associated variants, including six common and two low-frequency (minor allele frequency < 1%) [18]. The inclusion of founder populations in this meta-analysis facilitated the discovery of protein-coding risk variants in *NCSTN*, *PSENEN*, and *WNT10A. TMED10* was also prioritized as a candidate gene because its expression levels are associated with one of the risk variants. The four remaining regions contain noncoding variants. This study newly implicates Wnt/β-catenin signaling pathways and suggests a role for epidermal differentiation in HS pathogenesis [18]. Notably, the HS-protective allele at the *WNT10A* locus corresponds to a missense variant previously associated with hair follicle miniaturization in children and adults [89]. *WNT10A* is also a cause of monogenic ectodermal dysplasias [90,91].

An analysis of the Veterans Affairs Million Veterans Program (MVP) data included 4959 HS cases among 597,819 MVP participants and detected two genome-wide significant loci, replicating *SOX9* and identifying a novel variant near *HLA-DRB1* [92], for the first time implicating the HLA Class II region in HS pathogenesis.

Another GWAS of 4540 HS cases and over one million controls identified 11 significant associations across seven loci, including four previously reported loci, four novel signals within known regions, and three newly implicated loci [61]. Overlapping loci included *SOX9*, *KLF5*, the HLA region, and a noncoding region on chromosome 9. Novel associations were reported near *SUCNR1*, *NIN* (European and Admixed American-specific), and *MXRA7* (male-specific) [61]. Functional prioritization demonstrated allele-specific regulatory effects, with the HS risk allele at *KLF5* exhibiting markedly increased transcriptional activity and risk alleles at *SOX9* showing reduced activity [61]. Long-range enhancer evidence in mesenchymal cells implicates *KLF4* at the noncoding locus on chromosome 9.

The most recent GWAS, comprising 6361 cases and over 1.5 million controls, identified 12 independent loci, including four previously reported loci, including the *SOX9*, *KLF5*, and HLA loci and the chromosome 9 locus linked to *KLF4* by Broadway et al. Interestingly, this study prioritized several other genes at the chromosome 9 locus because their expression levels were previously associated with HS risk variants and they are differentially expressed in HS lesional skin compared to healthy skin (*FRRS1L*, *CTNNAL1*, *TMEM245)* [19]. Bioinformatic prioritization highlighted several genes across the loci, including *INAVA*, *CYP1B1*, *CXCR4*, *HLADRB1*, *KLF5*, *SOX9*, and *RBPP8*, among others. Ancestry-specific meta-analyses showed that *IL7R* was specific to European ancestry and *GNPDA2* was specific to African American ancestry. Integration of genetic associations with epigenomic and transcriptomic data revealed cell type-specific regulatory programs, including an epithelial gene program characterized by upregulated *SOX9*, *CXCR4*, and *CD74*. CXCR4-expressing keratinocytes mapped to aberrant epithelial structures in HS skin. Pharmacological inhibition of CXCR4 attenuated inflammatory signaling and WNT pathway activity while upregulating apoptosis in HS keratinocytes. Interestingly, CD74 undergoes γ-secretase-mediated processing, suggesting a mechanistic link between common-variant risk and rare γ-secretase loss-of-function variants previously implicated in familial HS. This study also identified an inverse genetic correlation between HS and male pattern hair loss, extending prior observations involving *WNT10A* and highlighting shared but divergent follicular regulatory pathways [19].

Collectively, these findings demonstrate that HS risk is strongly influenced by common genetic variation that converges on epithelial differentiation, immune signaling, and follicular biology.

**Table 4:** Summary of GWASs in HS.

GWAS	Year	Number of Cases	Loci *	Cohorts
Sun [60]	2023	1683 (discovery cohort); 290 (replication cohort)	*KLF5*, *SOX9*	UNC Chapel Hill HS ProCARE, UK Biobank, FinnGen, BioVU,
Andersen [18]	2025	4814	*NCSTN*, *HLA Class II*, *PSENEN*, *WNT10A*, *TMED10*, *KLF5*, *SOX9*, *chr. 9*	Danish Blood Donor Study, Copenhagen Hospital Biobank, Zealand University, Icelandic HS patient registry, UK Biobank, FinGen, Intermountain Inspire Registry, HerediGene
Wendland [92]	2025	4959	*HLA Class II*, *SOX9*	VA’s Million Veteran Program (MVP)
Broadaway [61]	2025	4540	*SUCNR1*, *HLA Class II*, *KLF4 (chr. 9)*, *KLF5*, *NIN* ^‡^, *SOX9*, *MXRA7* ^†^	HS Program for Research and Care Excellence (ProCARE)
Khan [19]	2025	6361	*KIF21B*, *CYP1B1*, *CXCR4*, *HLA Class II*, *FRR1SL (chr. 9)*, *KLF5*, *KLF12*, *FOS*, *SOX9*, *RBBP8*, *MAPRE2*, *KREMEN1*, *IL7R* ^‡^, *GNPDA2* ^‡^	eMERGE-III, University College Dublin, CHOP_GSA array, CHOP-HM, Michigan, Penn, Estonian biobank, MVP, AoU, FinGen, Hunt

* Loci identified by nearest gene. † Male-specific. ‡ Population-specific.

The Khan et al. GWAS also developed and evaluated a genome-wide, polygenic risk score (PRS), a quantitative measure that aggregates the effects of common risk variants to estimate individual genetic susceptibility to HS [19]. Each standard deviation increase in the PRS was associated with a 1.4-fold increased HS risk, and individuals in the top 1% of the PRS distribution had more than eightfold higher odds of HS compared with the remaining population [19]. Given the clinical heterogeneity of HS and its frequent early misclassification as recurrent abscess or pilonidal disease, particularly in acute care settings, such tools may eventually have adjunctive value in risk stratification among individuals with suggestive presentations [1]. More broadly, integration of genetic risk with clinical features represents a potential future approach to prioritizing referral and prompting earlier diagnostic consideration of HS [1,93,94]. However, these translational applications remain speculative at present, and further studies are needed to determine the clinical utility, calibration, and generalizability of HS PRS.

Several important limitations currently constrain the clinical implementation of HS polygenic risk scores [59]. As with PRS in other complex diseases, predictive performance varies substantially across ancestral groups, particularly when derived from discovery cohorts that are not ancestrally diverse, underscoring the need for validation in independent and multi-ancestry populations. In addition, external validation and assessment of model calibration will be essential before HS PRS can be considered for broader application. Even if predictive performance proves robust, the optimal integration of PRS into existing diagnostic pathways remains uncertain, including how genetic risk information would be combined with clinical features to improve recognition of early or atypical disease. Finally, there are currently no prospective clinical studies demonstrating that the use of HS PRS improves diagnostic accuracy, shortens time to diagnosis, or meaningfully changes patient outcomes in routine practice. Accordingly, although HS PRS represents a promising area of investigation, its practical clinical utility remains to be established.

Across studies, individual GWASs identify partially distinct risk loci, reflecting differences in sample size, ancestry composition, and study design. A notable strength of the current literature is that cohorts are largely non-overlapping, underscoring the opportunity for larger meta-analyses (Table 3). Combining cohorts across studies is expected to substantially improve statistical power, refine locus localization, and enhance biological and clinical interpretability. Importantly, genetic discovery in complex disease does not plateau with increasing sample size [58]. In breast cancer, for example, GWASs involving more than 130,000 cases have identified over 170 independent susceptibility loci [95]. Continued expansion and meta-analysis of HS cohorts will yield additional loci and deeper insight into disease biology.

### 3.5. Intersection of Rare and Common Variants

Several loci identified in GWASs correspond to genes implicated in Mendelian diseases, as summarized in Table 5. Integration of common and rare variant data provides complementary and clinically actionable information [96,97,98,99]. Common variant associations frequently implicate regulatory mechanisms, pathways, and disease-relevant cell types, whereas rare penetrant variants often directly identify causal genes and clarify the functional consequences of gene perturbation. Together, this convergence refines causal inference and highlights genes whose modulation is both biologically meaningful and therapeutically tractable. The number of successful targets with both rare penetrant variants and common risk variants implies that innate variation in function is an important characteristic of a promising drug target. Importantly, the presence of both types of variants yields the genetic equivalent of a dose–response curve. Common variants show the effects of subtle phenotypic changes, whereas rare, highly penetrant variants show the effects of strong perturbation, providing preclinical evidence for the effects of therapeutic modulation before exposing humans to drugs [99]. Importantly, many of these genes have not yet been associated with the clinical features of HS. Large-scale analyses of drug development pipelines have demonstrated that targets with human genetic support have approximately two- to threefold higher rates of clinical success than targets without such evidence, independent of allele frequency or effect size [96,97].

### 3.6. Summary of Genetic Findings and Future Directions

Together, human genetic studies indicate that HS risk is influenced by a continuum of inherited genetic variants, from rare, high-penetrant variants to common, highly polygenic variants. While γ-secretase genes represent the strongest rare-variant signal, further studies are needed to understand the loss-of-function mechanism. The implication of γ-secretase loss of function in regulating epithelial inflammation by a recent HS GWAS opens up a new line of inquiry. Candidate gene studies highlight biologically plausible pathways but largely lack definitive genetic support. GWASs currently represent the most scalable and informative approach to understanding HS genetics. Future progress will depend on larger, ancestrally diverse cohorts, cross-study meta-analyses, integration of functional genomics, and translation of genetic findings into clinically meaningful frameworks.

## 4. Discussion

Many individuals with comparable risk profiles (either genetic or environmental) never develop HS, while others develop severe disease despite limited genetic or environmental burden. This clinical heterogeneity underscores a role for both the genome and environment in shaping disease risk. In addition, HS rates appear to be increasing, though it is unclear whether increased recognition and diagnosis or extrinsic factors are driving this increased prevalence. Family history is strongly implicated in patients with HS; however, not all family members experience HS, which suggests that other factors may be at play.

Current evidence supports a multifactorial model of HS pathogenesis in which genetic predisposition interacts with environmental exposures to determine whether the disease manifests, its age of onset, its severity, and how it responds to various interventions. In this framework, environmental factors act as modifiers of risk on a genetically susceptible background rather than as sole causal agents. Understanding the genetic architecture of HS therefore provides critical insight into why shared exposures produce divergent clinical outcomes and helps contextualize environmental risk factors within a biologically grounded model of disease.

Human genetic research offers a principled framework for understanding why environmental risk factors do not exert uniform effects across individuals [100]. By resolving etiologic heterogeneity, genetic studies distinguish shared exposure from differential biological susceptibility and can clarify the mechanisms through which environmental factors influence disease risk [101]. In complex diseases, genetic evidence has been shown to contextualize epidemiologic associations, prioritize biologically relevant pathways, and identify which environmental exposures are likely to be causal rather than correlational [100]. Framing HS genetics within this paradigm reinforces the view that environmental risk factors act through genetically encoded homeostatic and inflammatory pathways rather than serve as sole drivers of disease.

Furthermore, the identification of environmentally responsive pathways, including xenobiotic metabolism (*CYP1B1*), microbial responses (*INAVA*, *CXCR4*, *CYP1B1*), and WNT signaling [20,21], suggests that genetic susceptibility likely modulates individual responses to environmental exposures.

There are challenges, however, to collecting environmental data to determine the true effect on genetics and disease pathogenesis. Research shows HS is associated with factors such as obesity, smoking, microbiome dysbiosis, dietary factors, and plastic consumption, but direct causality is difficult to confirm as patients encounter many environmental exposures over the course of their lifetimes. More research is needed to determine the direct effect of environmental factors and how genetics play a role. One future direction could be exploring the effects of dietary interventions on pathogenesis and disease severity.

Given the well-documented genetic and environmental influences on HS pathogenesis, HS should be viewed not as a purely genetic or environmental disease but as a condition arising from their interaction.

## 5. Conclusions

The recent literature suggests that both genetic and environmental factors contribute to the pathogenesis of HS. The increasing rates of HS diagnoses could be explained by either contributions of environmental factors or increased recognition of this disease. Obesity, smoking, microbiome dysbiosis, dietary factors, and plastic consumption are associated with disease pathogenesis and severity. In addition, the literature provides strong evidence of genetic factors predisposing a patient. The debate between the exposome and the genome is warranted. Leading research suggests that pathogenesis is at the intersection between genetic predisposition and environmental factors. Future studies should focus on the interaction between genes and the environment to better understand these interactions.

## Figures and Tables

**Table 1:** Environmental risk factors associated with HS pathogenesis.

Environmental Factor	Association with HS	Reference
Obesity	Increased BMI associated with increased disease severity	Schrader, 2014 [22]
Adipose Tissue	Increased sex hormones and possible friction	Hetemaki, 2021 [23]
Hormonal Imbalance	Increased hormones influencing homeostasis of the skin	Chu, 2021 [24]
Inflammatory Markers	Increased in obese patients, possibly contributing to etiology	Malara, 2018 [25]
Mechanical Stress	Increased adiposity leading to mechanical stress on the skin	Boer, 2021 [26]
Metabolic Abnormalities	Increased risk of HS with metabolic abnormalities	Malara, 2018 [25]
Smoking	Increases inflammation and promotes colonization of Staphylococcus aureus	Acharya, 2020 [27]
Microbiome	Increased anaerobes and decreased normal skin bacteria	Riverain-Gillet, 2020 [3]
Dietary Factors		
Ultra-Processed Foods	Strongly correlated with HS prevalence	Haddad, 2024 [4]
Simple Carbohydrates	Activation of mTOR and androgen receptors	Shen, 2023 [5]
Dairy	Activation of mTOR and androgen receptors	Shen, 2023 [5]
Plastic Consumption	Inhibit Nicastrin (NCSTN) protein expression promoting inflammation	Williams, 2025 [6]

**Table 2:** Overview of genetic study designs used to identify, characterize, and interpret the contribution of genetic variation to human disease. Each approach addresses distinct clinical or biologic questions and differs in its sensitivity to rare or common variation.

Study Design	Clinical or Translational Application	Primary Question Addressed	Predominant Variant Type
Family-based linkage analysis	Gene discovery in large pedigrees with Mendelian inheritance	Which genomic region segregates with disease in families (is present in cases and absent in related controls)	Rare, high-penetrance variants
Candidate gene association study	Hypothesis-driven testing of biologically or clinically plausible genes	Is variation in a specific gene associated with disease	Common or rare
Whole exome sequencing (WES)	Gene discovery	Which rare protein altering variants explain disease; does any gene in the genome contain more variants in cases compared to controls	Rare
	Diagnostic analysis	Phenotype-driven method to test a specific hypothesis regarding established disease genes	
Genome wide association study (GWAS)	Gene discovery	Which common variants across the genome increase disease risk	Common
Polygenic risk score (PRS)	Risk prediction in individuals	What is an individual’s cumulative genetic risk based on common variants	Common
	Compare polygenic architectures across diseases	Is genetically predicted disease A associated with a clinical outcome B	

**Table 3:** Strength of genetic and functional evidence supporting candidate gene categories implicated in HS. Summary of the major classes of candidate genes reported in association with HS, including γ-secretase complex genes, inborn errors of immunity, and ectodermal dysplasia-associated genes. For each category, the strength of human genetic association evidence, degree of replication across independent cohorts, availability of case series or case reports, and extent of functional genomics validation are assessed. Importantly, levels of evidence vary substantially across gene categories, with robust familial segregation and replication observed for γ-secretase genes, contrasted with largely descriptive or syndromic evidence for immune and ectodermal genes, highlighting key gaps in HS gene–disease validation.

Candidate Gene Category	Strength of Genetic Association Evidence	Replication Across Independent Cohorts	Case Series/Case Reports	Functional Genomics Evidence
γ-Secretase complex genes (e.g., *NCSTN*, *PSENEN*)	Strong. Multiple pedigrees demonstrate autosomal dominant inheritance with segregating loss-of-function variants; statistically robust.	Strong. Replicated across many unrelated families and population of different ancestries.	Large multigenerational families and aggregated familial case series	Limited and indirect. A small number of studies demonstrate γ-secretase loss-of-function, leading to dysregulated Notch signaling and secondary PI3K–AKT pathway activation; not systematically replicated in HS patient tissue.
Inborn errors of immunity (IEI) (e.g., *PSTPIP1*)	Weak to moderate. No consistent gene-level association signal; enrichment inferred from syndromic overlap rather than statistical association.	No formal replication at the gene or variant level.	Case reports and small case series describing HS as part of auto-inflammatory or immunodeficiency phenotypes	Underutilized. Many IEI genes have gold-standard functional assays in immunology; however, studies reporting HS comorbidity rarely perform functional validation in HS patient samples.
Ectodermal dysplasia (ED) genes (e.g., *GJB2*)	Weak. Largely based on phenotypic co-presentation; no disease-specific statistical enrichment.	Mostly single reports or very small cohorts.	Case reports and limited case series describing HS phenotypes in ectodermal syndromes	Sparse and indirect. Functional work largely from mouse or developmental biology models; minimal direct functional genomics in HS tissue.

**Table 5:** Mendelian diseases in HS GWASs: common variant risk loci.

Common Variant Loci Identified in HS GWASs	Pathway/Function	Mendelian Disease Genes (OMIM ID)
*SOX9*	Epidermal differentiation and follicular inflammation	OMIM: 278850 and 616425 (46,XY sex reversal) OMIM: 114290 (Acampomelic and campomelic dysplasias)
*NCSTN*	Gamma secretase signaling	OMIM: 142690 (Autosomal dominant HS)
*PSENEN*	Gamma secretase signaling; Notch signaling	OMIM: 613736 (Autosomal dominant HS)
*WNT10A*	HF development; NF-κB activation	OMIM: 257980 (Ectodermal dysplasia) OMIM: 224750 (Schopf–Schulz–Passarge syndrome) OMIM: 150400 (Tooth agenesis, selective)
*CYP1B1*	Xenobiotic metabolism; estrogen metabolism; oxidative stress	OMIM: 231300 (Primary congenital glaucoma) OMIM: 617315 (Anterior segment dysgenesis)
*CXCR4*	Chemokine signaling; immune cell trafficking; stem cell homing	OMIM: 193670 (WHIM syndrome)
*RBBP8*	DNA damage response; cell cycle control	OMIM: 606744 (Seckel syndrome) OMIM: 251255 (Jawad syndrome)
*PSMB8*	Immunoproteosome; MHC class I antigen generation	OMIM: 256040 (Proteasome-associated auto-inflammatory syndrome 1)
*PSMB9*	Immunoproteosome; MHC class I antigen processing and presentation	OMIM: 620796 (Proteasome-associated auto-inflammatory syndrome 6)
*KREMEN1*	Wnt/β-catenin signaling inhibition; hair follicle morphogenesis; ectodermal development	OMIM: 617392 (Ectodermal dysplasia)

## Data Availability

No new data were created or analyzed in this study.

## Abbreviations

The following abbreviations are used in this manuscript:

BMI	Body mass index
CD	Cluster of differentiation
CTNNAL	Catenin alpha-like
CXCR	C-X-C motif chemokine receptor
CYP	Cytochrome P450
DNA	Deoxyribonucleic acid
ED	Ectodermal dysplasia
FRRS	Ferric chelate reductase
GJB	Gap junction beta
GNPDA	Glucosamine-6-phosphate deaminase
GWASs	Genome-wide association studies
HLA	Human leukocyte antigen
HLA-DRB	Human leukocyte antigen-DR beta
HS	Hidradenitis suppurativa
IEI	Inborn errors of immunity
IKBKB	Inhibitor of nuclear factor kappa B kinase subunit beta
IL	Interleukin
INAVA	Innate immunity activator
IRF	Interferon regulatory factor
KDF	Keratinocyte differentiation factor
KID	Keratitis–ichthyosis–deafness
KIF	Kinesin superfamily
KLF	Kruppel-like factor
KREMEN	Kringle-containing transmembrane protein
LACC	Lactose domain-containing
MAPRE	Microtubule-associated protein RP/EB family E
MHC	Major histocompatibility complex
mTOR	Mechanistic target of rapamycin
MVP	Million Veterans Program
MXRA	Matrix remodeling-associated
NCSTN	Nicastrin
NIN	Nodule inception
OMIM	Online Mendelian inheritance in man
p-EDs	Plastic-associated endocrine-disrupting chemicals
PAPA	Pyogenic sterile arthritis, pyoderma gangrenosum, and acne
PI3K–AKT	Phosphoinositide 3-kinase–protein kinase B
PRS	Polygenic risk score analysis
ProCARE	Program for Research and Care Excellence
PSEN	Presenilin
PSENEN	Presenilin enhancer, gamma-secretase subunit
PSTPIP	Proline–serine–threonine phosphatase interacting protein
PSMB	Proteasome subunit beta
RBBP	Retinoblastoma-binding protein
RBPP	Reactivity-based protein profiling
SOX	SRY-related HMG box
STAT	Signal transducer and activator of transcription
SUCNR	Succinate receptor
TMED	Transmembrane emp34 domain-containing protein
TMEM	Transmembrane
UK	United Kingdom
US	United States
WES	Whole exome sequencing
WGS	Whole genome sequencing
WHIM	Warts, hypogammaglobulinemia, infections, and myelokathexis
Wnt	Wingless/integrated
XIAP	X-linked inhibitor of apoptosis
